# Photocatalytic decontamination of phenol and petrochemical wastewater through ZnO/TiO_2_ decorated on reduced graphene oxide nanocomposite: influential operating factors, mechanism, and electrical energy consumption†

**DOI:** 10.1039/c8ra07936f

**Published:** 2018-11-30

**Authors:** Farzan Hayati, Ali Akbar Isari, Moslem Fattahi, Bagher Anvaripour, Sahand Jorfi

**Affiliations:** Department of Chemical Engineering, Abadan Faculty of Petroleum Engineering, Petroleum University of Technology Abadan Iran farzan.hayati73@gmail.com aliichemeng@gmail.com fattahi@put.ac.ir anvaripour@put.ac.ir; Department of Environmental Health Engineering, School of Health, Ahvaz Jundishapur University of Medical Sciences Ahvaz Iran sahand369@yahoo.com; Environmental Technologies Research Center, Ahvaz Jundishapur University of Medical Sciences Ahvaz Iran

## Abstract

ZnO/TiO_2_ anchored on a reduced graphene oxide (rGO) ternary nanocomposite heterojunction was synthesized *via* the multi-step method including hydrothermal, solvothermal and sol–gel methods. XRD, Raman, FESEM, EDX, Dot Mapping EDS, BET, FTIR, UV-VIS, TGA, and EIS techniques were utilized for characterizing as-synthesized catalysts. The XRD and Raman data proved the formation of anatase phase TiO_2_ and wurtzite phase ZnO in the prepared samples. Further, the UV-Vis spectrum confirmed that the band gap value of ZnO/TiO_2_ diminished on introduction of graphene oxide. Photocatalytic performance of the fabricated catalysts was investigated by decontamination of phenol in aqueous solutions. The effect of different operational factors such as pH, catalyst dosage, phenol concentration, and light illumination was investigated to find the optimum decontamination conditions. According to the results, complete degradation of phenol was achieved at pH = 4, catalyst dosage of 0.6 g L^−1^, light intensity of 150 W, and phenol initial concentration of 60 ppm at 160 min under visible light illumination. With the addition of graphene oxide to the composite, a significant increase was detected in the photocatalytic performance due to the higher available surface area and lower electron/hole recombination rate. In addition, the scavenging experiments revealed that the ·OH is responsible for the degradation of phenol during the reaction. The degradation mechanism, economic performance, mineralization, and recyclability were also investigated. Kinetic studies confirmed that photocatalytic degradation process followed the pseudo-first-order kinetic model. A case of real wastewater treatment was used to examine the performance of the catalyst for real case studies.

## Abstract 2

ZnO/TiO_2_ anchored on a reduced graphene oxide (rGO) ternary nanocomposite heterojunction was synthesized *via* the multi-step method including hydrothermal, solvothermal and sol–gel methods.

## Graphical abstract

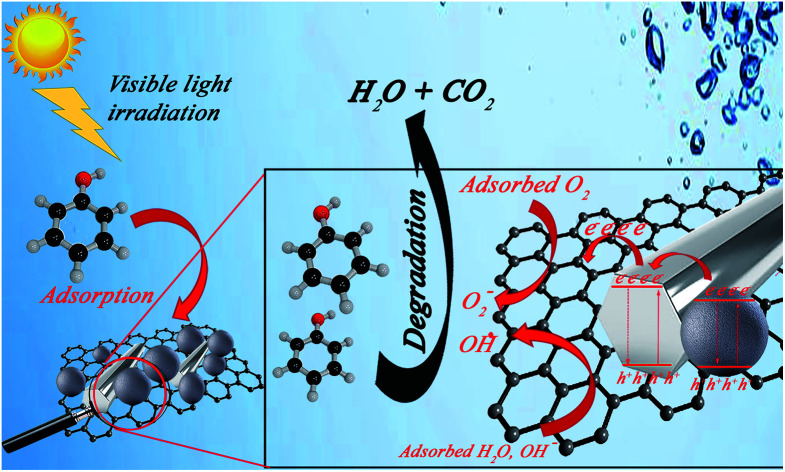

## 1 Introduction

Nowadays, providing clean and healthy water resources has become one of the most important concerns of governments due to the population growth, considerable increase in water withdrawal, and production of large amounts of wastewater by different industries. Many industries produce water effluent containing organic compounds which are considered harmful environmental contaminants. The discharge of these contaminants makes changes to the ecosystem of aqueous systems by reducing the concentration of dissolved oxygen in natural channels. Petrochemical plants, petroleum refineries, and textile industries are the most important sources of phenol and heavy aromatic compounds. Phenol and phenolic compounds are bio-recalcitrant toxic organic compounds which are very harmful to the environment. Based on the European Union regulation no. 80/778/EC, the maximum allowable concentration of phenolic compounds is 0.5 mg L^−1^ in drinking water. Accordingly, wastewater containing a high concentration of aromatic and toxic contaminants should be effectively treated before being discharged into natural channels.^1–6^

In particular, due to non-biodegradability of phenolic compounds in aqueous solutions, conventional biological methods are inefficient for effective treatment of these compounds. In recent years, advanced oxidation processes (AOPs) have changed into an alternative solution in the wastewater treatment plants for degradation of recalcitrant organic compounds thanks to their high degradation efficiency, low-cost, fast reaction rate, and non-selective degradation. AOPs are based on production of very reactive radicals involving ozonation, H_2_O_2_/O_3_ combined with UV, semiconductor photocatalysis, Fenton, photo-Fenton, ultrasound irradiation and wet air oxidation.^3,7–9^ Amongst AOPs, heterogeneous semiconductor photocatalysis has been proven as one of the most appropriate and effective methods for degradation of recalcitrant organic contaminants in water thanks to its high performance, low material consumption, and cost-effectiveness. Recently, many semiconductors have been under usage as photocatalyst such as TiO_2_, ZnO, WO_3_, MgO, SiO_2_, Fe_2_O_3_, and CdS.^10–16^ Semiconductor photocatalysts have recently demonstrated high photocatalytic activity for decontamination of organic compounds in aqueous solutions. Typically, titania has been revealed as a promising catalyst and has been widely used in photocatalytic degradation of organic contaminants due to its electrochemical properties, low cost, availability, high stability and non-toxicity. However, pristine TiO_2_ has a fast electron/hole (e/h) recombination rate which restricts the photo-generated electron–holes lifetime. Further, these semiconductors have a large band gap and low efficiency in degradation of contaminants under visible light regions. Alternatively, several techniques have been chosen to improve the efficiency including metal/nonmetal doping, hetero-structures, multi-component composites using other semiconductors, architectural control, *etc.* Combination of wide bandgap semiconductors with narrow bandgap semiconductors and formation of type I heterojunctions can enhance the photocatalytic activity of titania.^17–25^

The composite of TiO_2_ and ZnO has shown to have a higher photocatalytic activity than pristine TiO_2_ and/or ZnO because of type II hetero-junction formation which inhibits the e/h recombination rate. Furthermore, the heterogeneous structure formation by two semiconductors extends the photo response of composites to visible light area.^26–30^

Co-adsorbents such as zeolites, carbonaceous materials, and nano-clays are another way for further enhancing the photocatalytic activity and adsorption capacitance of TiO_2_. Recently, graphene nanosheets have been used as an effective ESI† due to its favorable properties like solar radiation absorption, separation of efficient charge carriers, exposed reactive sites, and high surface area. Graphene has superior properties such as optical transparency, high surface area, and high electrical conductivity along with other physiochemical properties. It was shown that this support can enhance the degradation efficiency through making a hybrid by semiconductor metal oxides. Many heterogeneous nanostructure photocatalysts coupled with graphene nanosheets have been synthesized and developed in recent years.^17,19,26,31–34^

Photocatalytic process efficiency is significantly affected by electron–hole recombination rate which could be controlled by various parameters such as interfacial properties, electronic properties, chemical composition, and physical dimension.^23^ Various statements have been suggested about the characteristics of graphene in hetero-structure nanocomposites which included graphene nanosheets in their frameworks: (I) formation of Zn–O–C, Zn–C, and Ti–O–C bonds by graphene defect states improves the solar or visible light adsorption capacity of catalysts; (II) graphene can accept or donate electrons due to its high electron mobility in order to reduce the e/h recombination rate of ZnO/TiO_2_ nanocomposite, thus prolonging the life-time of excited electrons during the reaction; (III) graphene enhances the adsorption capacity of nanocomposites and creates more reactive sites for sorption of target contaminants because of its high BET specific surface area.^23,35,36^ However, graphene nanosheets are hydrophobic, making it challenging to disperse graphene in aqueous solutions for preparing homogenous solutions. Homogeneous dispersion of graphene oxide (GO) in aqueous mediums is easier than that of graphene due to its functional groups such as –OH and –COOH. Therefore, GO nanosheets are commonly used in the synthesis of photocatalytic nanocomposites. Thus, we expected that the introduction of rGO in the TiO_2_/ZnO framework would resolve the TiO_2_/ZnO restrictions and considerably enhance the photocatalytic activity of TiO_2_/ZnO under visible light illumination. To the best of our knowledge, many studies have explored enhanced photocatalytic activity of titania *via* ZnO and/or graphene. Shahbazi *et al.* used TiO_2_ nanoparticles over graphene nanosheets synthesized in hydrothermal process as a photocatalyst for degradation of phenol in aqueous media under UV irradiation.^3^ Raliya *et al.* used TiO_2_, ZnO, GO/TiO_2_, GO/ZnO, TiO_2_/ZnO for mineralization of methyl orange dye under visible light illumination.^20^ Nuengmatcha *et al.* evaluated the sonocatalytic performance of ZnO/G/TiO_2_ nanocomposite for degrading dye contaminants under ultrasonic irradiation.^17^ Malekshoar *et al.* used TiO_2_/graphene, ZnO/graphene and their physical mixture for degradation of phenol with sun light irradiation.^26^ Liu *et al.* synthesized ZnO/TiO_2_/rGo nanocomposite *via* microwave-assisted method and evaluated its photocatalytic performance by reduction of Cr(vi).^27^

Due to low quantum yield of bare TiO_2_ and its wide band gap value, ZnO as a coupling semiconductor material is able to suppress the electron/hole recombination rate of TiO_2_ and improve its photocatalytic activity. Besides, reduced graphene oxide was used for surface modification of titania and improving its adsorption as well as charge conductivity performance. To the best of our knowledge, there are limited studies which used couple of modifications for degradation of phenol and real petrochemical wastewater under visible light irradiation. In this study, catalysts were synthesized *via* multi-step method involving hydrothermal, solvothermal and sol–gel methods. The prepared composite was characterized and studied for phenol photocatalytic degradation under visible light illumination in aqueous solutions. The effect of different operational parameters including pH, phenol concentration, catalyst dosage, and light illumination were examined. In addition, stability of fabricated nanocomposite during reactions, mineralization rate, economic estimation of the process, possible degradation mechanism, and trapping studies were also investigated. In the end, a petrochemical wastewater was treated *via* the fabricated catalyst.

## 2 Materials and methods

### 2.1 Materials

GO and graphene was purchased from US Research nanomaterial (USA). Titanium(iv) butoxide (TB; 99.9%) was utilized as the precursor of TiO_2_. Also, zinc nitrate hexahydrate (ZN) was employed as the precursor of ZnO. Other chemicals were purchased from Merck (Germany). All materials were utilized without further purification because of their analytical grade.

### 2.2 Real wastewater sampling

The real petrochemical wastewater was collected from wastewater effluent of Maroun Petrochemical Company, Mahshahr, Iran. The collected samples were stored in 10 L containers at 4 °C. The physiochemical features of the collected wastewater such as COD, TDS, BOD, TSS, and pH were determined based on specific standard techniques (ISO 5667-10:1992).

### 2.3 Preparation of TiO_2_

For this purpose, 10 mL of TB was added to 40 mL of absolute ethanol (AE) and sonicated for 30 min (mixture 1). Another solution containing 10 mL ethanol, 80 mL deionized water (DI) and 3 mL HNO_3_ was added to mixture 1 dropwise under constant stirring. After 1 h of stirring, the homogeneous solution was heated at 80 °C in a hot water bath for 30 min. Then, the hot solution was allowed to cool and form the gel. Next, the gel remained still for 24 h, which was then dried in an oven at 100 °C for 8 h. At the end, the fine powder of dried gel was annealed at 400 °C for 4 h to achieve the anatase phase of TiO_2_(Ti).^33,37^

### 2.4 Preparation of ZnO

To this aim, 10 gr ZN was dissolved in 250 mL DI water and sonicated for 2 h. The prepared solution was heated at 90 °C under constant stirring for another 5 h. Next, the obtained mixture was dried at 100 °C overnight. Finally, the obtained fine powder was annealed at 600 °C for 2 h to achieve the wurtzite phase of ZnO.^38^

### 2.5 Preparation of rGO/ZnO/TiO_2_

rGO/ZnO/TiO_2_ (GZnTi) was synthesized through facile hydrothermal method. A suitable amount of GO (0.05, 0.075 and 0.1 gr GO for 5, 7.5 and 10 wt%, respectively) was dissolved in a mixture containing 20 mL DI water and 40 mL AE and sonicated for 1 h. Then, 0.1 g of ZnO and 0.9 g TiO_2_ (10% ZnO/90% TiO_2_) were added to the prepared mixture and sonicated for another 2 h to attain a homogeneous suspension. The attained suspension was transferred to 100 mL Teflon sealed hydrothermal autoclave and maintained at 120 °C for 3 h to develop the nanocomposite of rGO, ZnO and TiO_2_. Finally, the achieved composite was filtered and washed with ethanol several times and dried at room temperature. ZnO/TiO_2_ (ZnTi) nanocomposite was synthesized by the mentioned hydrothermal method without adding GO.^39^

### 2.6 Characterization

Horiba-Jobin-Yvonlabram-HR UV-VIS-NIR Raman spectrometer (*λ*_laser_ = 532 nm) was used for Raman spectroscopy. Fourier transform infrared (FT-IR) spectra of the synthesized photocatalysts were evaluated using Bruker-VERTEX70 apparatus. The X-Ray Diffraction (XRD) profile of the synthesized samples was recorded by X-Ray diffractometer (Quantachrome, NOVA 2000) through graphite monochromatic CuK_α_ irradiation (0.15406 nm wave length) within the range of 10–80° with 0.05° step size. The field emission scanning electron microscope (FESEM), Energy Dispersive X-ray (EDX) and Dot-Mapping of samples were recorded using Mira-3-Taksan operating at 15.0 kV. Brunauer–Emmett–Teller (BET) specific surface areas of the samples were determined by N_2_-adsorption–desorption isotherms at 77 K using Beckman-Coulter 3100 instrument. Barrett–Joyner–Halenda (BJH) model was used for determining the pore mean diameter using N_2_-adsorption measurement with 0.99 bar relative pressure. UV-Vis Diffuse reflectance spectroscopy (UV-VIS DRS) of specimens was examined using a SHI-MADZU spectrometer model UV-1240 (ranging from 200–900 nm with BaSO_4_ as reference). The electrochemical impedance spectroscopy (EIS) of the synthesized catalysts was investigated *via* potential-galvanostat PGSTAT302N instrument in a three-electrode-cell using saturated calomel electrode and platinum foil as the reference electrode and counter electrode, respectively. AC voltage amplitude, frequency range, and electrolyte were 10 mV, 10–100 kHz, and NaCl 3.5 wt%, respectively. The total organic carbon (TOC) of the degraded solution was studied *via* the DC-190 of Rosemount-Dohrmann analyzer. Thermogravimetric analysis (TGA) of the as-synthesized nanostructures was performed by a Q-600 TA-apparatus under air flow at a warming rating of 5 °C min^−1^ ranging from ambient temperature to 800 °C.

### 2.7 Photocatalytic activity

To prevent any ion interference during the reaction, DI water was used for preparing all solutions. In order to evaluate the photocatalytic activity of as-synthesized photocatalysts, phenol degradation (Ph-degradation) in aqueous medium was used as the objective contaminant. In the reaction process, three visible 270.7 Cd lamps equipped with *λ* > 420 nm filter was utilized as the visible light source, and the experiments were performed at the ambient temperature. First, a 250 mL solution was prepared with a certain amount of phenol. Then, a specific amount of as-synthesized catalyst was added to the phenol solution. With addition of NaOH 0.5 M or HCl 0.5 M L^−1^, the pH of the solution was adjusted. The pH of the media was determined using S210-Std-Kit Mettler-Toledo pH-meter under 250 rpm stirring. To achieve adsorption–desorption equilibrium, the mixture was loaded on to a 250 mL reactor and stirred (at 250 rpm and 27 ± 1 °C) for 60 min in the dark. The photocatalytic reaction was initiated by exposing the prepared suspension to visible light irradiation.

At specific time periods, 5 mL of the solution was sampled to determine the phenol concentration. PIT320 Universal centrifuge apparatus was used to remove the photocatalysts from the aqueous solution. The phenol concentration of the sample was calculated by UV2100 Unico UV-visible spectrophotometer to record the specific absorption at *λ*_max_ = 270 nm (maximum absorption wavelength).^3^ The removal and mineralization efficiencies of the system were calculated using the following equations:1
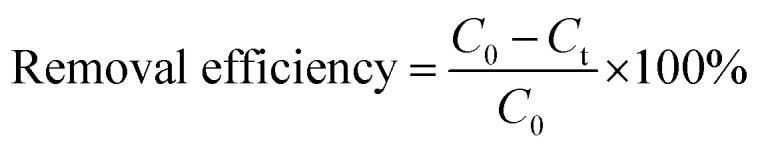
2
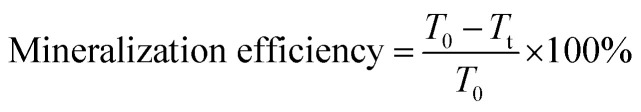
where, *C*_0_ and *C*_t_ represent the concentration of phenol at the initial time and sampling time. *T*_0_ and *T*_t_ denote TOC of the solution at the initial time and sampling time, respectively.

The adsorption capacity of the system was obtained through the following equation:3
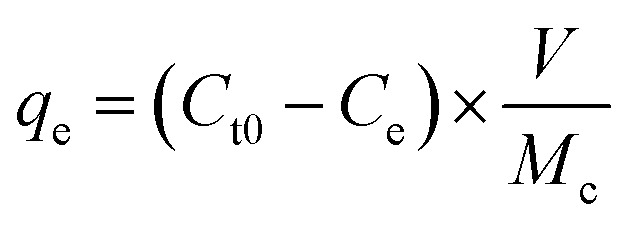
where *C*_t0_ was initial phenol concentration (mg L^−1^), *C*_e_ is the concentration of phenol at the adsorption equilibrium state (mg L^−1^), *V* is volume of the mixture (L) and *M*_c_ is the weight of GZnTi (g).

## 3 Results and discussion

### 3.1 Characterization of photocatalysts

#### 3.1.1 BET

**Fig. 1:**
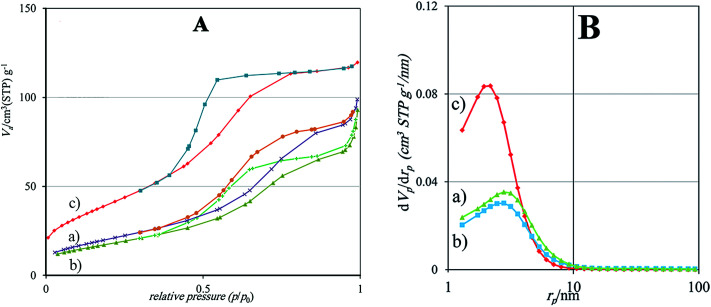
(A) Nitrogen adsorption–desorption isotherms of (a) Ti, (b) ZnTi and (c) GZnTi; (B) BJH patterns of (a) Ti, (b) ZnTi and (c) GZnTi samples.

Fig. 1A and B illustrate nitrogen adsorption–desorption isotherms and BJH patterns of Ti, ZnTi and GZnTi samples. According to IUPAC classification, N_2_ adsorption/desorption isotherms belong to type IV with H_4_ type hysteresis loops at *P*/*P*_0_ ranging around 0.03–0.99, suggesting the presence of abundant mesoporous structures in all samples. This isotherm is used for porous materials and often seen for industrial catalysts. BET surface areas of Ti, ZnTi and GZnTi were found to be 76.805, 66.112 and 149.95 m^2^ g^−1^, respectively. The obtained results indicated elevated BET surface area of GZnTi, compared with ZnTi, which is an effect of graphene addition. This phenomenon revealed that graphene is an efficient support for ZnTi photocatalyst thanks to enhancing the adsorption capacity of the catalyst. From BJH patterns, it is clear that the pore diameter distribution of catalysts was around 5 nm, representing the mesoporous structure of the samples.^1^

#### 3.1.2 XRD

**Fig. 2:**
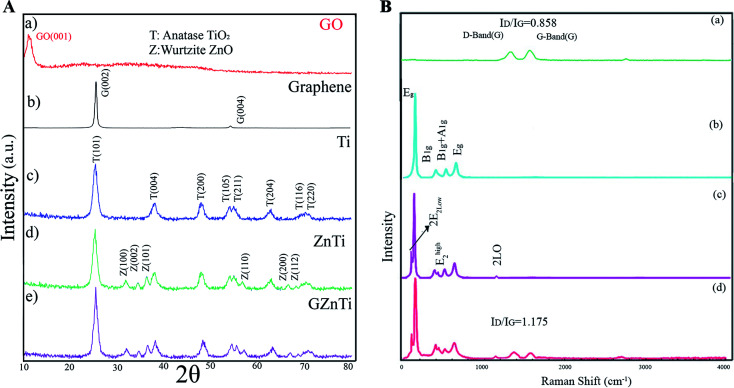
(A) XRD patterns of (a) GO, (b) graphene, (c) Ti, (d) ZnTi, and (e) GZnTi; (B) Raman spectra of (a) graphene, (b) Ti, (c) ZnTi, and (d) GZnTi.

XRD is a well-known and powerful method for structural analysis of nanocomposites, and has been used by researchers since 1950s. Fig. 2A displays the XRD patterns of Ti, ZnTi and GZnTi. As can be seen in Fig. 2A, the characteristic peaks at 2*θ* = 25.35, 38.00, 47.56, 54.15, 54.95, 63.05, 69.02 and 70.40 correspond to (1 0 1), (0 0 4), (2 0 0), (1 0 5), (2 1 1), (2 0 4), (1 1 6), and (2 2 0) planes respectively. These peaks represent pure anatase phase of titania according to JCPDS (21–1272). With introduction of ZnO, new peaks were observed at 2*θ* = 31.80, 34.75, 36.40, 56.8, 66.70 and 68.25 corresponding to wurtzite planes of (1 0 0), (0 0 2), (1 0 1), (1 1 0), (2 0 0), and (1 1 2) respectively, according to JCPDS (36–1451). Further, the ZnTi pattern reveals that the addition of ZnO does not make any changes in the TiO_2_ crystallite structure, and its remain in anatase phase. The major phase of anatase and wurtzite can be seen from the GZnTi pattern, revealing the presence of TiO_2_ and ZnO in the composite, respectively. This finding suggests that GO preserved the crystalline structure of the composite. The characteristic peak observed at 2*θ* = 11.04° of GO pattern matched (0 0 1) facet of GO. This peak could not be seen in the GZnTi pattern because GO was reduced during the thermal process.^40,41^ However, it is difficult to recognize the characteristic peak of graphene in the GZnTi profile. The reason can be related to the fact that the characteristic peak of graphene located at 2*θ* = 25.1° overlapped with the characteristic peak of TiO_2_ at 2*θ* = 25.35° (see Fig. 2A). Furthermore, the average grain sizes of as-prepared samples were estimated by the Debye–Scherrer's equation. The crystalline size of TiO_2_ for Ti, ZnTi and GZnTi samples was found to be 8.59, 6.68, and 6.31. The obtained results reveal that the addition of GO into the ZnTi composite considerably hindered crystalline growth.

#### 3.1.3 Raman

Fig. 2B represents Raman spectra of Ti, ZnTi, GZnTi, and GO samples. Raman-modes of anatase TiO_2_ including E_g_, B_1g_, B_1g_ + A_1g_, and E_g_ were observed at 147.78, 397.89, 525.78, and 639.53, respectively for all catalysts. Also, in ZnTi and GZnTi Raman profiles, typical modes of ZnO were seen at 96.71, 434.75, and 1149.47 corresponding to 2E^low^_2_, E^high^_2_, and 2LO wurtzite active-modes of ZnO, respectively. The obtained results are in good agreement with XRD reports. Furthermore, compared with the Raman profile of Ti, ZnTi and GZnTi patterns shifted to higher wavenumbers. This could be ascribed to distortion of titania due to incorporation of ZnO or GO. Raman shifts of GO and GZnTi indicate new peaks at 1354 and 1576, matching D-band and G-band of carbon materials contained in the mentioned samples. Meanwhile, *I*_D-band_/*I*_G-band_ ratio is an efficient tool for determining the functionalization degree of samples and defects in carbonaceous materials. The *I*_D-band_/*I*_G-band_ ratio of GZnTi increased to 1.175 compared to that of GO which was found to be 0.858. This suggests successful reduction of GO to rGO during the thermal process, which is in excellent accordance with previous reports.^26,42^

#### 3.1.4 FTIR

**Fig. 3:**
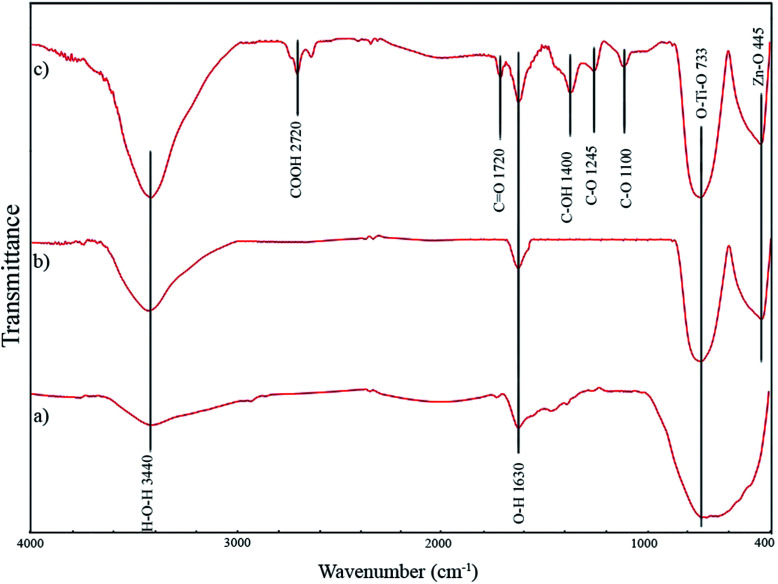
FTIR spectra of (a) Ti, (b) ZnTi, and (c) GZnTi.

Fig. 3 indicates the FTIR spectra of Ti, ZnTi, and GZnTi. For all FTIR patterns, a wide band located within the range of 630–845 cm^−1^ is observed, which corresponds to Ti–O–Ti stretching vibration of the interconnected octahedral [TiO_6_]. This indicates the presence of titania in all catalysts. Notably, there is a new peak around 541 cm^−1^ in the ZnTi and GZnTi samples, associated with the Zn–O stretching vibration bonds, demonstrating existence of ZnO in ZnTi and GZnTi nanocomposites. Furthermore, the broad peaks at 1630 and 3440 are attributed to –OH and H–O–H vibration bonds, respectively, suggesting presence of hydroxyl groups and moisture adsorbed on the catalyst surface. Interestingly, the mentioned peaks for Ti are weaker because of limitations for adsorption of water in this catalyst. As can be seen in the FTIR diagram of GZnTi, bonds at 3440, 2720,1720, 1630, 1400, 1245, 1100 are related to H–O–H stretching, COOH vibrations, C=O vibrations, O–H vibrations, C–OH stretching vibrations, C–O stretching vibration, and C–OH stretching vibration, respectively. The formation of the mentioned carbon-containing bonds in GZnTi pattern revealed existence of GO in the ZnTi nanocomposite.

#### 3.1.5 UV-DRS

**Fig. 4:**
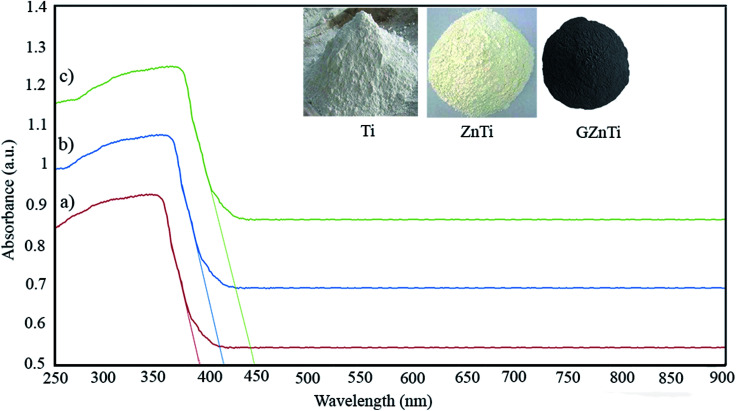
UV-visible adsorption spectra of (a) Ti, (b) ZnTi, and (c) GZnTi.

The optical properties of as-prepared photocatalysts were examined *via* UV-DRS analysis. Fig. 4 represents the UV-visible adsorption spectra of Ti, ZnTi, and GZnTi within the range of 200–900 nm. As can be observed, all of the samples have had intense adsorption in UV-light area (200–400 nm), suggesting their activation within this range. Notably, the adsorption intensity of GZnTi in visible-light region (400–700 nm) has been considerably higher than that of Ti and ZnTi. The inset of Fig. 4 shows the colors of photocatalysts. According to this figure, incorporation of GO into the ZnTi host changes the color of the catalyst from light (white for Ti and ZnTi) to dark (gray for GZnTi). Thus, darker catalyst color represents more intense adsorption. A slight positive shift of adsorption edge was caused by the interaction of ZnO with the titania framework. Furthermore, the adsorption edge of GZnTi nanocomposite had a red shift toward higher wavenumbers for 27 nm than that of ZnTi sample, since GO layers have successfully interacted with ZnTi nanoparticles. Hence, GO and ZnO are able to enhance the light adsorption intensity and optical response of titania across visible regions, which are favorable for the photocatalytic efficiency of systems on industrial scales. The band gap values of specimens were determined using Planck equation as follows:4
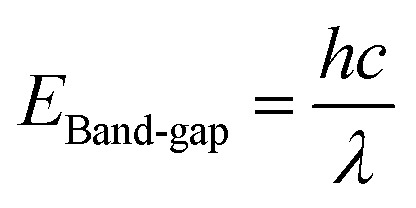
where, *λ* is the cut-off wavelength, *c* represents the speed of light (2.99 × 10^8^ m s^−1^), and *h* denotes the Planck constant (4.13566 × 10^−15^). The band gap calculation details of the samples are listed in Table 1. As can be observed, the band gap value of GZnTi was lower than that of Ti and ZnTi. The obtained results can be attributed to the characteristic light adsorption potential of GO contained in the nanocomposite and to high rate of charge migrations between the semiconductors (titania and ZnO) and rGO layers.^43^

**Table 1:** Band gap and cut of wavelength of prepared samples

Sample	Cut of wave length (*λ*) (nm)	Band gap (eV)
Ti	385	3.21
ZnTi	419	2.95
GZnTi	446	2.77

#### 3.1.6 FESEM

**Fig. 5:**
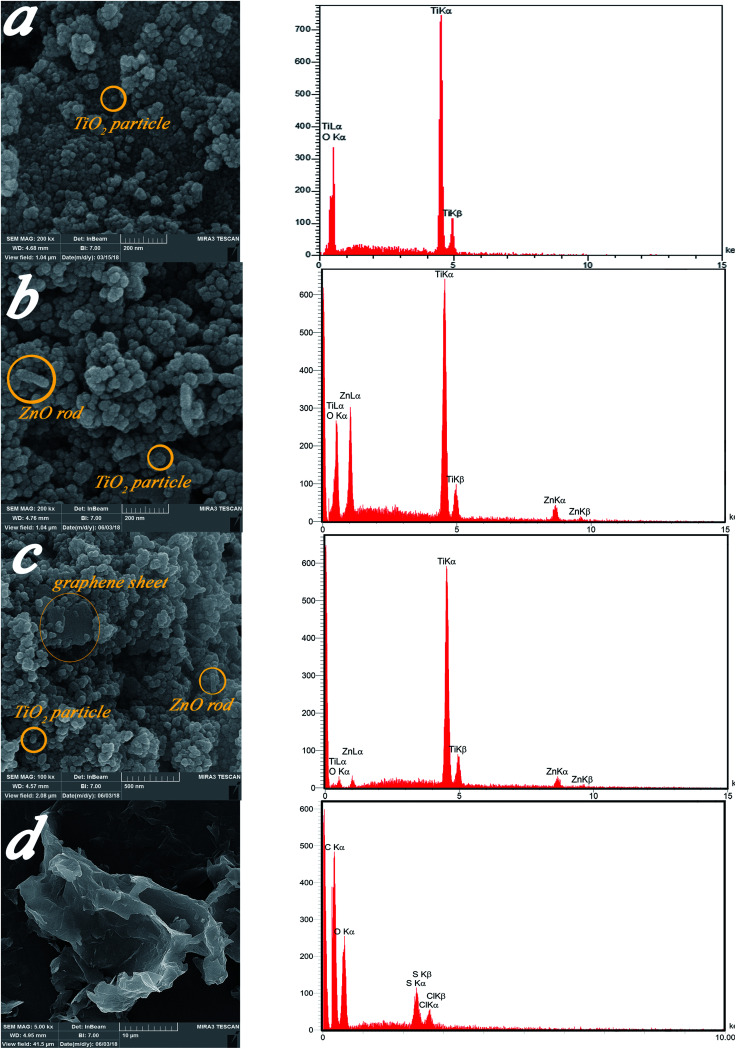
FESEM micrographs and of (a) Ti, (b) ZnTi, (c) GZnTi, and (d) GO with corresponding EDX tests.

Fig. 5(a–d) illustrates the FESEM micrographs of Ti, ZnTi, GZnTi and GO with corresponding EDX tests. As can be seen in Fig. 5a and b, neat TiO_2_ nanocomposite appeared with uniform sphere-shaped morphology and a trace content of rod-shaped ZnO was detected in ZnTi sample. In this regard, similar morphologies has been obtained in the literature. This indicates that introduction of ZnO preserved the initial morphology of titania. Fig. 5c and d demonstrate the FESEM micrographs of GZnTi and GO, respectively. The flat plane-like morphology of GO has been presented in Fig. 5c and d. Also, the interaction of titania and ZnO with GO can clearly be observed in Fig. 5c. Furthermore, the EDX profiles of specimens revealed major peaks of Ti, O, Zn, and C, confirming their high purity. According to the obtained results listed in Table 2, the theoretical weight composition of elements are a little different with the EDX results for each synthesized sample. The reason is that the EDX is merely a local analysis from the entire surface of samples, so the obtained errors are acceptable.^44^

**Table 2:** Weight composition of elements in the synthesized samples

Sample	Element	Calculated weight percent (wt%) for synthesis process	Obtained weight percent (wt%) from EDX
Ti	Ti	59.9	58.29
	O	40.1	41.71
ZnTi (10% ZnO, 90% TiO_2_)	Ti	53.91	52.79
	O	38.05	38.23
	Zn	8.03	8.98
GZnTi (7.5% rGO, 10% ZnO, 82.5% TiO_2_)	Ti	49.41	47.64
	O	35.06	36.12
	Zn	8.03	9.23
	C	7.5	7.01
GO	C	Not need	59.62
	O	Not need	35.72
	S	Not need	3.04
	Cl	Not need	1.63

**Fig. 6:**
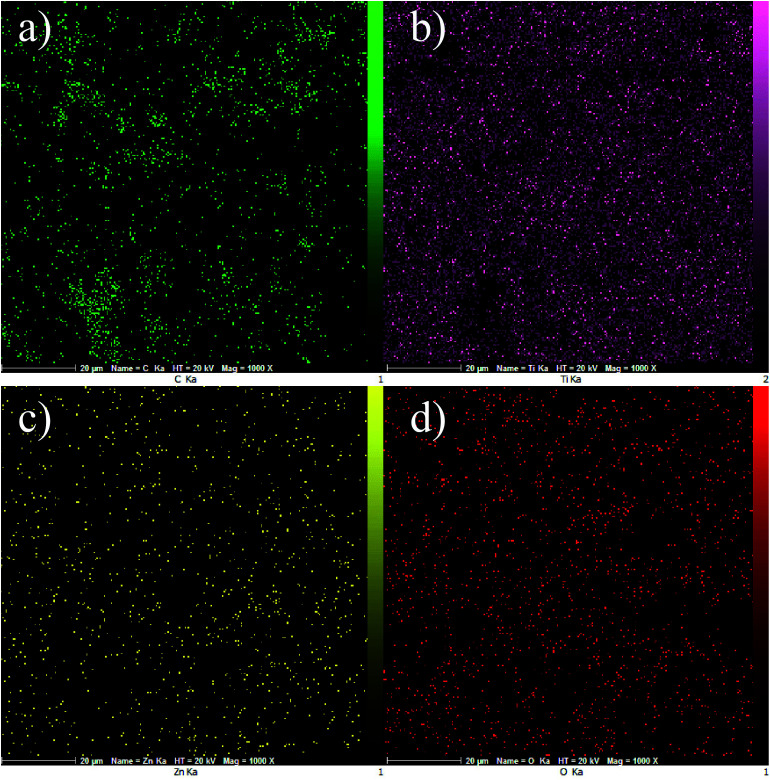
Dot-mapping of GZnTi photocatalyst (a) carbon, (b) titanium (c) zinc, and (d) oxygen.

Fig. 6a–d display EDS mapping images of the GZnTi photocatalyst. The obtained findings reveal homogeneous distribution of elements on the surface of the GZnTi photocatalyst.

#### 3.1.7 TGA

**Fig. 7:**
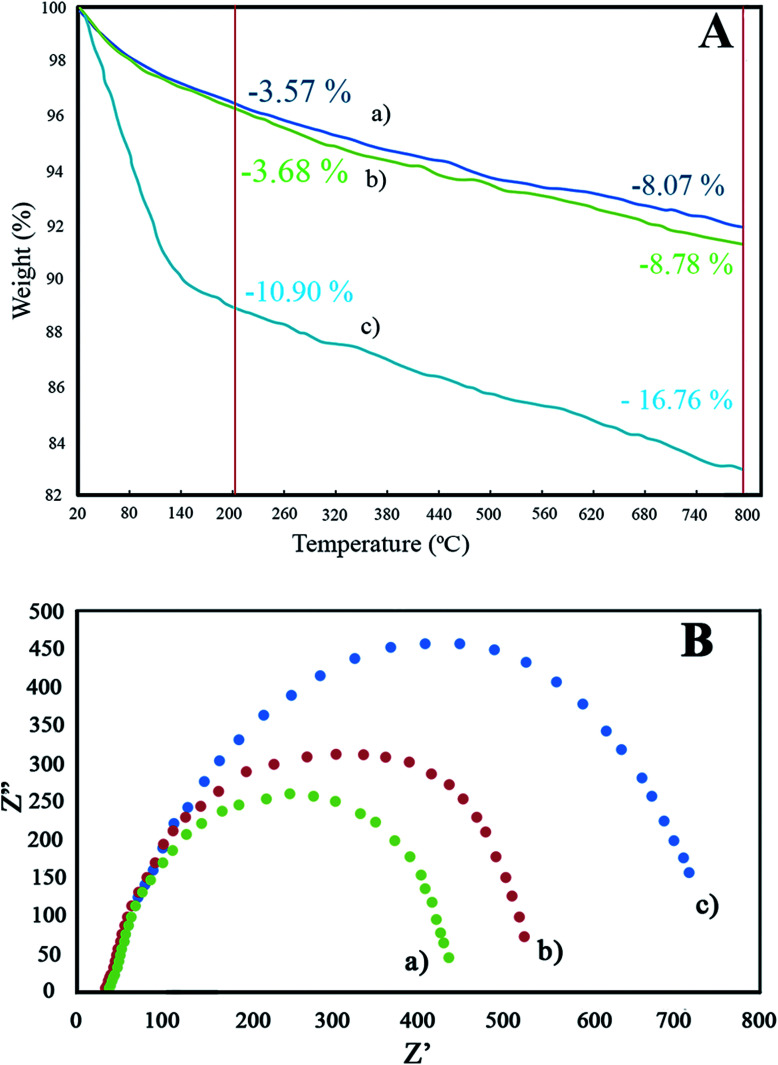
(A) TGA patterns of (a) Ti, (b) ZnTi, and (c) GZnTi; (B) EIS spectra of (a) Ti, (b) ZnTi, and (c) GZnTi catalysts under visible light irradiation.

TGA was utilized to estimate the thermal stability and quantity of RGO contained in GZnTi nanocomposite. The TGA patterns of Ti, ZnTi, and GZnTi are displayed in Fig. 7A. All patterns have a primary decomposition state starting from the ambient temperature to 200 °C, which is ascribed to evaporation of moisture adsorbed on the surface of samples. The order of the primary decomposition ratio of the samples was found to be GZnTi > Ti > ZnTi, which is in excellent accordance with BET results, where *S*_BET_ of GZnTi was greater than that of Ti and ZnTi. Thus, a higher moisture content could be adsorbed on the surface of catalysts.^1,3^ Notably, in the GZnTi profile, a secondary decomposition step was initiated from 200 °C to 800 °C, associated with combustion or decomposition of rGO contained in the GZnTi catalyst. The results indicate that the real weight ratio of rGO in the GZnTi nanocomposite is almost the same as the theoretical weight ratio of rGO before synthesis, confirming the accuracy of the synthesis process.

#### 3.1.8 EIS

The electrochemical properties of the samples were explored by powerful EIS spectra. Fig. 7B exhibits the EIS Nyquist diagrams of Ti, ZnTi, and GZnTi. EIS Nyquist represents three types of resistance; (i) contact resistance, (ii) intrinsic material resistance, and (iii) electrolyte resistance. The radius of the semicircle of Nyquist plots is related to the interfacial charge migration, which depends on the charge separation efficiencies of samples. As can be seen from Fig. 7B, the order of radii of semi-circles was found to be Ti < ZnTi < GZnTi, indicating that introduction of ZnO and (or) GO enhanced the electron migration rate of the sample, thereby improving the electron/hole separation rate of the GZnTi photocatalyst.^1^ Accordingly, the obtained EIS results suggest that the GZnTi photocatalyst is desirable for photocatalytic degradation reactions.

### 3.2 Photocatalyst performance

#### 3.2.1 Effect of GO content

The photocatalytic performance of GZnTi, ZnTi, and Ti composites was investigated to find the optimum concentration of GO in catalysts for the maximum Ph-degradation efficiency in the presence of visible-light irradiation at pH of 5, catalyst dosage of 0.8 g L^−1^, phenol concentration of 60 ppm. Ph-degradation rate of Ti was 40%, indicating low efficiency of pristine titania for decontamination of phenol under visible irradiation due to its high e/h recombination rate and wide band gap value. Also, with introduction of ZnO into TiO_2_, the Ph-degradation efficiency increased from 40% to 56%. This increase could be ascribed to the positive effect of ZnO on photo-excited electron trapping. In contrast to ZnTi, the Ph-degradation rate of all GZnTi nanocomposites grew, indicating that addition of GO considerably enhanced the photocatalytic performance of catalysts under visible light irradiation. Apparently, for the GZnTi nanocomposites, by increasing the weight percentage of GO from 5 to 7.5 wt%, the photocatalytic activity rose from 40% to 86.4%. However, by raising the GO concentration further to more than 7.5 wt%, the removal rate diminished to 73.5%. Mechanistically, GO can improve the adsorption capacity and reduce electron-hole-recombination-ratio, thereby enhancing the degradation efficiency.^23,45^ This effect was clearly observed in the case of GZnTi 5 wt% and 7.5 wt%, where the Ph-degradation efficiency increased considerably from 40% to 86.4%. Nevertheless, higher GO concentrations can scatter the light irradiations and reduce the available light adsorption surface of TiO_2_ and ZnO NPs, and compromise the photocatalytic performance.^45^ Accordingly, at high weight percentages of GO, TiO_2_, and ZnO, NPs aggregate on the surface of GO, thereby lessening the adsorption capacity of GO sheets and light adsorption of TiO_2_ and ZnO NPs. This, in turn, leads to lower photocatalytic performances. Accordingly, the GO concentration 7.5 wt% is an optimum concentration of GO in the GZnTi composite.

#### 3.2.2 Effect of pH

**Fig. 8:**
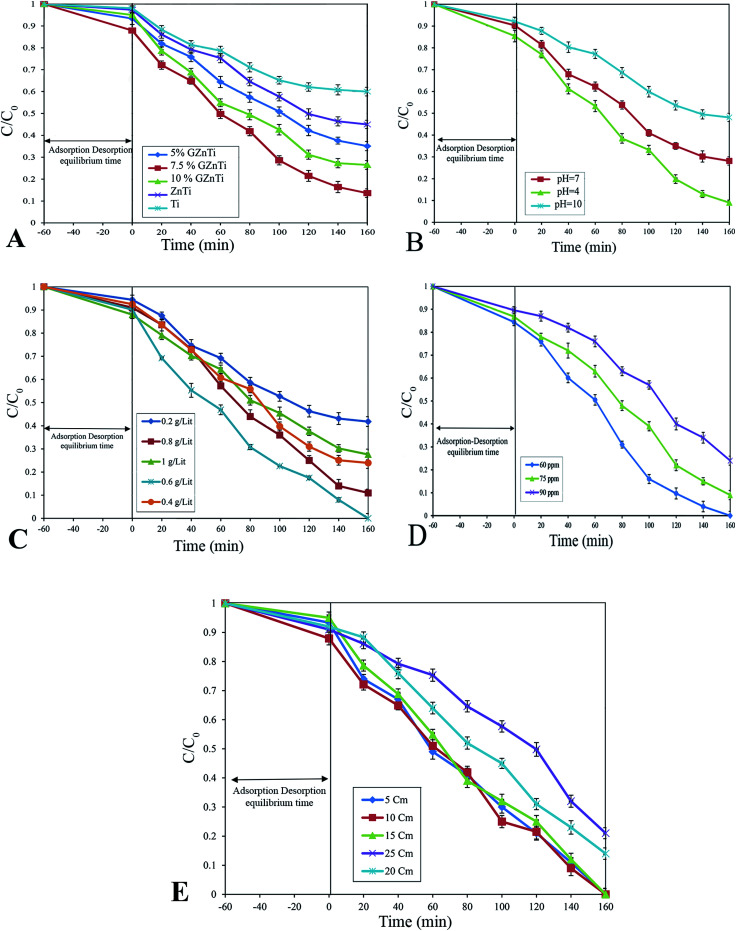
The effect of operational parameters on photocatalytic Ph-degradation; (A) effect of GO content at pH: 5, catalyst dosage: 0.8 g L^−1^, phenol concentration: 60 ppm; (B) effect of pH at catalyst dosage: 0.8 g L^−1^, phenol concentration: 60 ppm; (C) effect of catalyst dosage at pH: 4, phenol concentration: 60 ppm; (D) effect of phenol concentration at pH: 4, catalyst dosage: 0.6 g L^−1^; (E) effect of light source distance at pH: 4, catalyst dosage: 0.6 g L^−1^, and phenol concentration: 60 ppm.

Based on studies, the initial pH value of a solution is one of the important operational parameters which can influence the degradation rate of organic compounds during heterogeneous photocatalyst degradation. pH has a significant influence on the contaminant hydrolysis, catalysts' surface properties, oxidant, and contaminant ionization degree. The experiments were conducted at pH of 4–10, catalyst dosage of 0.8 g L^−1^, irradiation time of 160 min and phenol concentration of 60 ppm. As can be seen in Fig. 8B, the Ph-degradation in the acidic solution is higher than in neutral solution and alkaline solution, which dropped from 91% to 51.9% with pH rise from 4 to 10. These differences between degradation efficiencies at various pH ranges could be described by two reasons: (I) the oxidation ability of hydroxyl radicals is greater under acidic conditions; however, presence of HCO_3_^−^ and CO_3_^2−^ under alkaline conditions interferes in the reaction of hydroxyl radicals, thus reducing its oxidation potential.^46^ This validates that the Ph-degradation by photo-induced radicals is higher under acidic solutions than in alkaline solutions. (II) The low adsorption of negatively charged system components and interference of HCO_3_^−^ and CO_3_^2−^ (*i.e.* at pH value of 10) are the results of very low Ph-degradation rates. Therefore, effective Ph-degradation can occur within the range of 4–6.5 (lower than pH_ZPC_ in the acidic area). The results of degradation experiments revealed that the maximum Ph-degradation was 91% at pH = 4.

#### 3.2.3 Effect of GZnTi dosage

The amount of photocatalyst has a significant effect on the Ph-degradation efficiency. The effect of GZnTi photocatalyst was studied by varying the catalyst dosage. The experiments were performed at initial pH of 4, catalyst dosages of 0.2–1 g L^−1^, irradiation time of 160 min and phenol concentration of 60 ppm. The degradation rate of phenol for different catalyst dosages is shown in Fig. 8C. By raising the photocatalyst dosage from 0.2 g L^−1^ to 0.6 g L^−1^, more active sites were available and the specific surface area increased, resulting in enhanced Ph-degradation rate from 58.2% to 100%. Also, the superoxide and hydroxyl radical generation rate grew within this range. However, with addition of extra contents of the catalyst over 0.6 g L^−1^, the Ph-degradation rate declined from 100% to 72.4%. This could be resulted by: (I) lower solution transparency, light scattering and interception results due to excessive dosage of photocatalyst and prevention from light induction of some particles; (II) diminished pore volume and available surface area of the catalyst; (III) prevention of collisions between phenol and hydroxyl radicals; and (IV) excessive hydroxyl generation with higher doses of the catalyst. This recombines formation of H_2_O_2_ or reaction with O_3_ for production of HO_2_ radicals, which are less active than hydroxyl radicals.^7,19,47^

#### 3.2.4 Effect of phenol concentration

The effect of phenol concentration was studied at different phenol concentrations of 60–90 ppm, initial pH of 4, catalyst dosage of 0.6 g L^−1^, irradiation time of 160 min. Fig. 8D displays the obtained results for different phenol concentrations. As can be seen, the Ph-degradation efficiency is 76% at the concentration of 90 ppm, which significantly increases as the phenol concentration drops from 90 to 60 ppm. This can be ascribed to the fact that elevation of phenol concentration and its intermediate by-products deactivates more active sites and reduces the light penetration to active sites situated on the surfaces of catalysts, which are responsible for producing oxidative radicals. Also, at high phenol concentrations, a major portion of light illumination can be absorbed by phenol molecules and its intermediate by-products. This reduces the photocatalytic activity by limiting the light absorption capability of photocatalysts and lowering the concentration of ·O^−^ and ·OH.^19,46^ Herein, to a better assess, the catalytic activity of the synthesized GZnTi was compared with the detoxification of phenol reported in previous studies (see Table 3). GZnTi showed more photocatalytic performance because it indicated higher amounts of decontamination in less time under visible light than other catalysts. Therefore, it was concluded that the GZnTi nanocomposite was an efficient catalyst for decontamination under selected experimental parameters. It may be beneficial to use this nanocomposite for commercial applications due to its slight cost and great performance.

**Table 3:** Comparison of photocatalytic Ph-degradation results of this study with the open literature

Catalyst	Concentration (mg L^−1^) and volume (mL) of phenol	Catalyst amount g L^−1^	Degradation (%)	Illumination time (min)	Rate constant (min^−1^)	Ref.
GO/TiO_2_	14–100	1.48	100	180	—	3
rGO/TiO_2_	50–1700	—	96	180	0.0154	48
Pure TiO_2_	—	—	62	180	—	48
MWCNT–TiO_2_	50–800	1	96	300	0.0074	49
Pt–ZnO	15	—	>95	540	—	50
Fe–S–TiO_2_	20–60	1	99.4	600	—	51
CNT/Ce–TiO_2_	50–500	0.4	94	180	0.0012	6
BiPO_4_ (H_2_O_2_ assisted)	50–100	0.5 + 60 ppm H_2_O_2_	100	240	0.037	52
Fe_3_O_4_–ZnO	−200	0.325	82.8	150	0.0108	53
ZnO	50–200	1	69.75	120	0.015	54
ZnO	75–100	2.5	100	480	—	55
Co–Pd/BiVO_4_ (air flow through the suspension)	18.4–100	0.8	90	180	0.013	56
BiMoO_6_	20–100	1	90	480	0.0049	57
This study	60–250	0.6	100	160	0.0124	

#### 3.2.5 Effect of light distance

Position of light from the mixture plays a critical role to enhance the incident illumination intensity on the surface of the photoreactor. The effect of light distance on Ph-degradation was investigated within the range of 5–25 cm at pH of 4, catalyst dose of 0.6 g L^−1^, irradiation time of 160 min, and phenol concentration of 60 ppm. The results presented in Fig. 8E reveal that by increasing the light intensity from 5 to 15 cm, the Ph-degradation did not considerably change. This finding suggests that in the light distance range of 5–15 cm, sufficient content of photons delivered from the lamp to the photocatalyst surfaces for effective decontamination of organics in the process. However, at distances higher than 15 cm, there was a decrease in Ph-degradation rate which indicates the limitation in the light distance from the mixture in photocatalytic systems that in accordance with the ref. 58.

#### 3.2.6 Recyclability

**Fig. 9:**
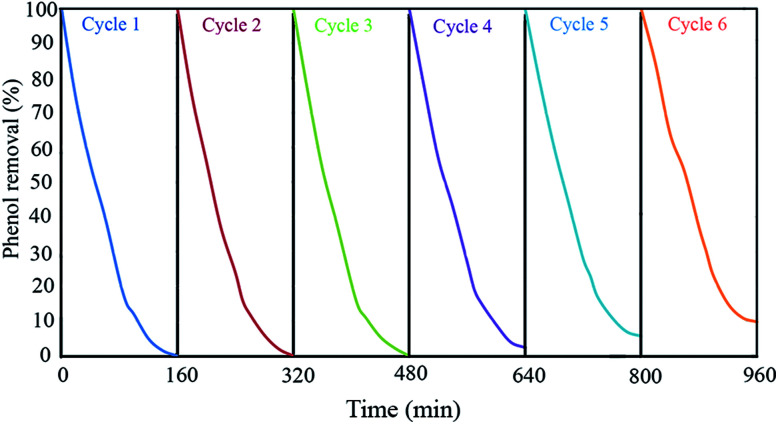
Recyclability of GZnTi in Ph-degradation process in the presence of visible light illumination through 6 cycles at pH: 4, catalyst dosage: 0.6 g L^−1^, and phenol concentration: 60 ppm (at the optimum Ph-degradation conditions).

In real application of heterogeneous photocatalysts in wastewater treatment plants, stability and recyclability of catalyst are absolutely important. The stability and recyclability of GZnTi were examined in a batch reactor under the optimum Ph-degradation condition (pH of 4, catalyst dosage of 0.6 g L^−1^, irradiation time of 160 min, and phenol concentration of 60 ppm). After each experiment, the catalyst was withdrawn from the solution and washed with water and ethanol mixture (2 : 1 volume ratio) to remove the phenol and its intermediate products on their pores and surfaces. Then, the mixture was dried at 80 °C for 2 h. This sequence was repeated for six times to find the Ph-degradation efficiency of each cycle. The results demonstrated in Fig. 9 indicate that the Ph-degradation efficiency was maintained within the range of 90–100% after six cycles, and showed good stability and recyclability for GZnTi. The photocatalytic activity reduction of GZnTi from 100% to 90.2% after six cycles could be explained by loss of catalyst during the washing process, shrinkage of the pore volume, and reduction of the BET specific area.^19^

#### 3.2.7 Trapping/radical scavengers

It has been proven that different reactive species including hydroxyl radical, hole, and superoxide radical are responsible for the oxidation of organic contaminants in aqueous solutions in photocatalytic degradation systems. The scavenger effect was studied under visible light irradiation, at pH of 4, catalyst dosage of 0.6 g L^−1^, irradiation time of 160 min, and phenol concentration of 60 ppm. For this aim, 10 mL of IPA (isopropyl-alcohol), BQ (*p*-benzoquinone), and EDTA 0.01 M (disodium ethylene diamine tetra acetic acid) was separately added to the photo-reactor as scavengers of hydroxyl radicals (·OH), superoxide radicals (·O_2_^−^), and holes (h^+^), respectively.^2,59^

**Fig. 10:**
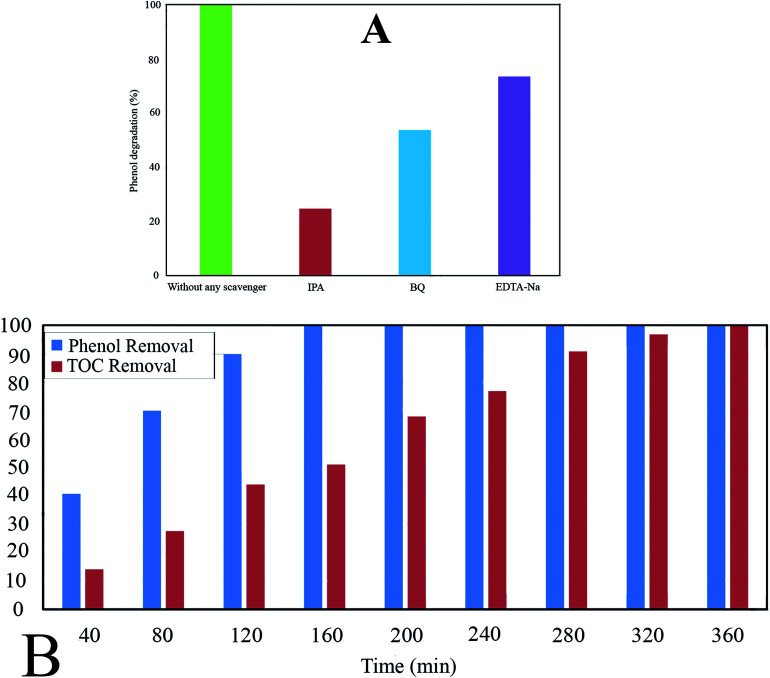
(A) Ph-degradation efficiency in the presence of different radical scavengers (IPA, BZQ, Na_2_-EDTA); (B) comparison of phenol removal and TOC removal at pH: 4, catalyst dosage: 0.6 g L^−1^, and phenol concentration: 60 ppm.

The Ph-degradation efficiency for different radical scavengers is exhibited in Fig. 10A. As can be seen, the Ph-degradation rates in the presence of ·OH, ·O_2_^−^ and h^+^ scavengers were found to be 24.75, 53.82, and 73.65, respectively. Accordingly, the dominancy order of species was: ·OH>·O_2_^−^ > h^+^, indicating that Ph-degradation process was mainly controlled by hydroxyl radicals.

#### 3.2.8 Mineralization

The mineralization rate of phenol was investigated through comparing TOC and Ph-degradation efficiency for different times under optimal conditions including pH of 4, catalyst dosage of 0.6 g L^−1^, and phenol concentration of 60 ppm.

The experimental results reveal that TOC removal rate rises to 100% after 360 min, while the Ph-degradation rate reaches 100% after 160 min (Fig. 10B). The difference between TOC and Ph-degradation can be ascribed to decomposition of phenol molecules to the intermediate by-products.

#### 3.2.9 Degradation kinetic

**Fig. 11:**
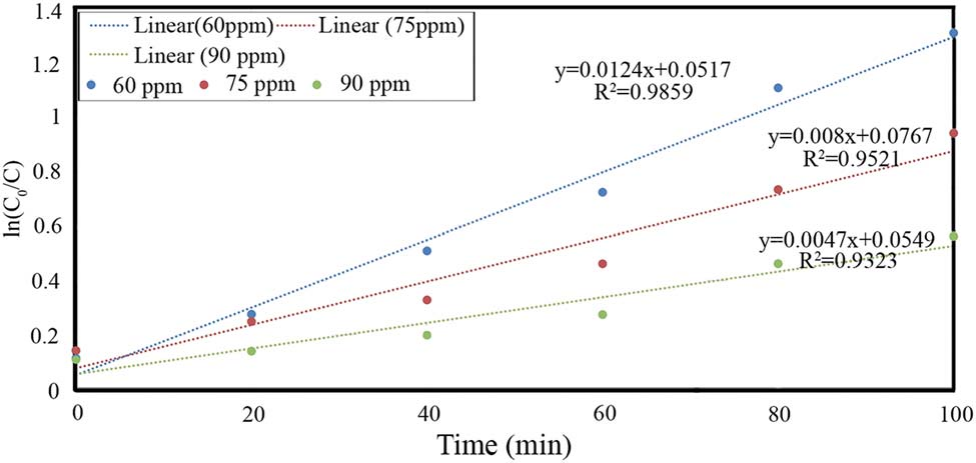
Pseudo-first order kinetic study of phenol for different phenol concentrations at pH: 4, catalyst dosage: 0.6 g L^−1^.

The pseudo-first order kinetic parameters of Ph-degradation kinetics were calculated by plotting ln(*C*_0_/*C*) *vs.* time for phenol concentrations of 60, 75 and 90 ppm under pH of 4, catalyst dosage of 0.6 g L^−1^, and irradiation time of 100 min. The rate constant and correlation coefficient were determined for different initial phenol concentrations and listed in Table 4. Based on the results, it can be established that the Ph-degradation kinetics at 60 ppm was best fitted by the pseudo-first-order reaction with 0.9859 regression coefficient and 0.0124 L min^−1^ rate constant (Fig. 11).^4^

**Table 4:** Pseudo-first order kinetic parameters of Ph-degradation kinetics

Kinetics model	Initial concentration (ppm)	Regression coefficient (*R*^2^)	Rate constant (min^−1^)
Pseudo first order, ln[*C*_0_/*C*] = −*kt*	60	0.9859	0.0124
	75	0.9521	0.008
	90	0.9323	0.0047

#### 3.2.10 Estimating electrical energy consumption

According to the kinetic results, decontamination rate of phenol *versus* time at different phenol concentrations matched pseudo-first-order kinetic model. On the other hand, heterogeneous photocatalysis technique is electricity intensive, so electric energy cost is one of the main portions of total costs. Thus, determination of simple figures of merit, which is based on electrical energy consumption, could be helpful and beneficial. The photochemistry commission of IUPAC for low-content contaminants and first-order reaction kinetic models suggested figures of merit by an equation for AOPs. According to this statement, the suitable figure of merit is the electrical energy per order (EEO), and introduced as the number of kW h of electrical energy necessary for reducing a contaminant content by one-order of magnitude (almost 90%) in 1000 L of contaminated water. The EEO 
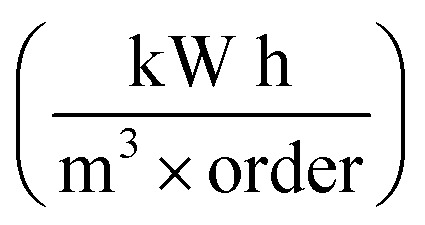
 of the system used in this investigation could be estimated from the following equation:^60^5
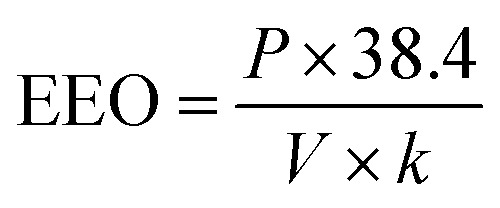
where, *P* is the rated power (kW) of the photocatalysis process, *V* represents the volume (L) of the solution contained in the photo-reactor, and *k* denotes pseudo-first order rate constant 
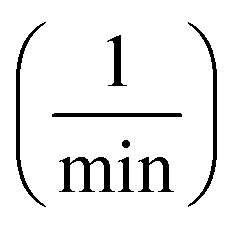
 of Ph-degradation kinetic model. EEOs of photocatalysis process for various phenol concentrations of 60, 75, and 90 ppm were found to be 0.46, 0.72, and 
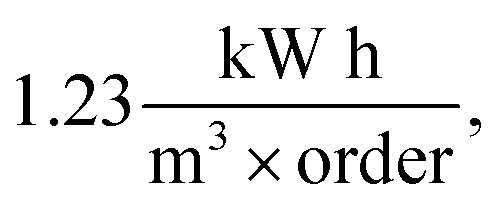
 respectively. Accordingly, the electrical energy price is 8.22 euro cent per kW h in European Nation market in 2017 for industrial plants. Hence, the contribution to price of water treatment regarding electricity consumption rate will be estimated as 3.82, 5.92, and 10.07 euro per m^3^, respectively. From the obtained calculations, it could be concluded that utilization of GZnTi for photocatalytic decontamination of phenol under visible irradiation is a low-cost and effective process compared to other conventional methods.

#### 3.2.11 Degradation mechanism

**Fig. 12:**
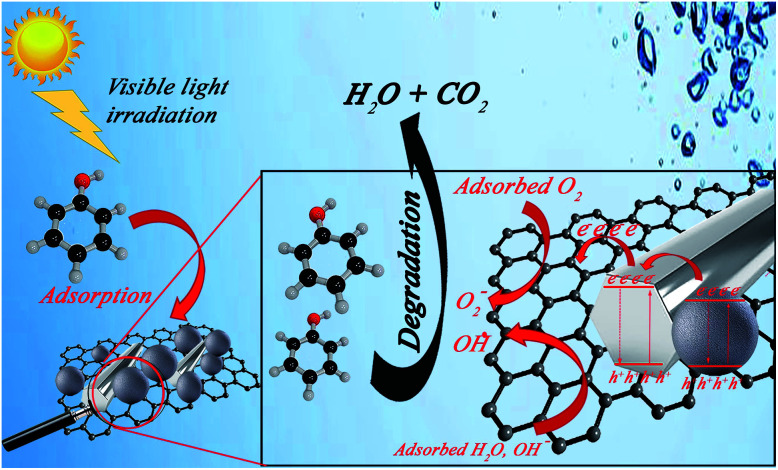
Tentative photocatalytic Ph-degradation mechanism under visible light irradiation using GZnTi.

Based on previous reports, the possible mechanism for photocatalytic Ph-degradation *via* GZnTi nanocomposite could be suggested as Fig. 12. As the nanocomposite is exposed to the visible light illumination, electrons in the valence band of titania migrate to the conduction band of ZnO. This process improves when rGO is used as a ESI.† Indeed, graphene acts as an excellent adsorbent for organic contaminants, sink for photo-excited electrons, and electron transporter to the composite surface. In this way, the effective roles of rGO nanosheets result in reduction of e/h recombination rate, thus enhancing photocatalytic performance of the nanocomposite.^38^

The created electrons and holes are trapped by O_2_ and H_2_O molecules, generating highly active peroxide and hydroxyl radicals, respectively. Therefore, the generated radicals attack the organic pollutants such as phenol molecules and oxidize them to intermediate by-products, which are finally mineralized to CO_2_ and H_2_O.^38^

#### 3.2.12 Real wastewater treatment

**Fig. 13:**
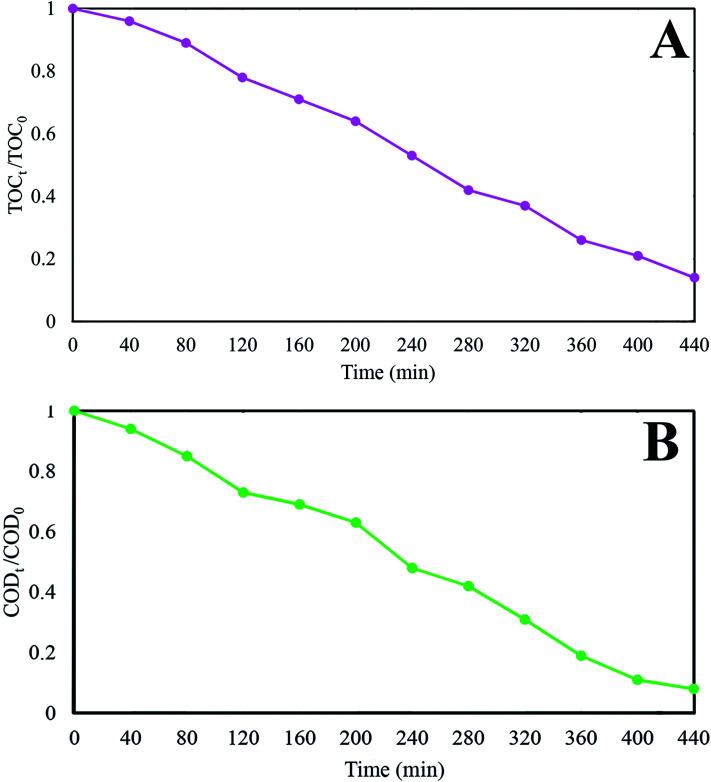
(A) TOC removal from a real petrochemical wastewater; (B) COD removal from a real petrochemical wastewater at pH: 4, catalyst dosage: 0.6 g L^−1^.

In order to evaluate the effectiveness of GZnTi nanocomposite in practical industrial uses, real petrochemical wastewater was utilized for treatment *via* GZnTi at pH = 4 and catalyst dosage of 0.6 g L^−1^ under visible light irradiation. The properties of the collected wastewater are listed in Table 5. The TOC and COD removal ratio of the wastewater as a function of time are presented in Fig. 13A and B. As can be seen, the TOC and COD removal ratio grew by prolonging the reaction time, which were found to be 85.9% and 92.1%, respectively, after 440 min. As a possible explanation, with prolongation of reaction time, more photons are adsorbed by the nanocomposite, leading to production of more oxidative radicals which are responsible for reducing TOC and COD of the wastewater during the process. To the best of our knowledge, some studies have reported similar findings for treating industrial wastewaters using AOPs.^4,61–63^

**Table 5:** The physiochemical wastewater properties

Parameter	Value range	Average
Total COD (mg L^−1^)	1135–1684	1410
BOD_5_ (mg L^−1^)	60–112	86
BOD_5_/COD	—	0.061
TOC (mg L^−1^)	737–1043	890
Turbidity (NTU)	11–19	15
TDS (mg L^−1^)	1309–1851	1580
TSS (mg L^−1^)	125–198	162
pH	6.6–7.8	7.2

#### 3.2.13 Adsorption studies

Adsorption has a key role in removal of organics in the photocatalytic systems because it determines the sorption mechanisms and also, reveals the different parameters impacting on the degradation rate. Interestingly, batch adsorption kinetics reports are useful for designing the industrial columns and scale-up applications. Noting that the experiments were performed at dark conditions, 25.6 °C, pH of 4, catalyst loading of 0.6 g L^−1^ and phenol concentration of 30–50 ppm. As could be seen in Fig. S1,† the adsorption rate of phenol (30 ppm) on GZnTi was very quick in the first stage of experiment (*t* < 30 min) which indicated the happening of a physical adsorption process. Finally, the adsorption process reached to the equilibrium adsorption state without any further phenol adsorption after 60 min which revealed the presence of a large number of reactive sites on the surface of the adsorbent saturated by phenol molecules during the process (Fig. S1†). In order to investigate the adsorption character and its performance, several experiments were carried out for kinetics and isotherms of phenol on the GZnTi photocatalyst. Three well-known kinetic models including intra-particle diffusion, pseudo first-order (PFO) and pseudo second-order (PSO) models and two isotherm models including Langmuir and Freundlich were considered for evaluation of experimental results. ESI† and constants of the aforementioned kinetics and isotherms as well as the corresponding regression coefficients (*R*^2^) are represented in Table 6. The correlation coefficient of intra-particle diffusion and PFO models were 0.9454 and 0.9121, respectively; whereas, the correlation coefficient of PSO model was expressed 0.9811. In other words, the obtained results from the kinetic studies revealed that the PSO model better describes the behavior of phenol adsorption on the GZnTi. It is noteworthy that PSO model demonstrates the fact that the chemisorption process is the dominant step in the adsorption experiment which occurred by donating or exchanging of electrons between phenol molecules and GZnTi binding sites. Furthermore, as given in Table 6, the *R*^2^ value for Langmuir model was greater than that of Freundlich model which indicates that the phenol adsorption followed Langmuir isotherm model. The maximum mono-layer adsorption capacity (*q*_max_) of 5.32 for phenol was calculated from Langmuir adsorption isotherm which demonstrated efficient adsorption of molecules on the surface of the GZnTi. As a result, GZnTi could be considered as an effective adsorbent for adsorption of aromatic compounds like phenol.^64–68^

**Table 6:** Constants of kinetics and isotherm models for phenol removal through adsorption GZnTi

Model	Value
**PFO**	
*K*_1_ (min^−1^)	0.0217
*q*_e cal_ (mg g^−1^)	7.62
*R* ^2^	0.9156

**PSO**	
*K*_2_ (mg g^−1^ min^−1^)	1.6366 × 10^−4^
*q*_e cal_ (mg g^−1^)	140.84
*R* ^2^	0.9513

**Intra-particle diffusion**	
*K*_id_ (mg g^−1^ min^−0.5^)	11.162
*R* ^2^	0.8358

**Freundlich**	
*N*	4.468
*K*_f_ (mg g^−1^ (L mg^−1^)^−1/*n*^)	55.37
*R* ^2^	0.8836

**Langmuir**	
*q*_max_ (mg g^−1^)	121.95
*K*_L_ (L mg^−1^)	0.322
*R* ^2^	0.9893

## 4 Conclusion

In this investigation, visible light active GZnTi ternary nanocomposites were successfully prepared with different weight ratios of GO. They were confirmed *via* XRD, Raman, FT-IR, BET, UV-Vis DRS, EIS, TGA, FESEM, EDX, and EDS analysis. According to the obtained findings, GO in the GZnTi nanocomposite was effectively reduced through the synthesis process. Furthermore, presence of ZnO nanorods was detected *via* FESEM micrographs. Compared to pure titania, within corporation of GO and ZnO, the band gap value diminished, BET surface area increased, and the recombination rate of charges dropped. The catalytic performance of as-prepared ternary nanocomposites was evaluated by Ph-degradation under visible light irradiation. According to the experiments, the GZnTi nanocomposite demonstrated complete degradation of phenol when the GO content was 7.5 wt%, pH value was 4, photocatalyst dosage was 0.6 g L^−1^, phenol concentration was 60 ppm, light intensity was 150 W, and reaction time was 160 min. Furthermore, based on the electrical energy consumption study, 3.81832, 5.9184, and 10.0739 euro/m^3^ were required for Ph-degradation with a concentration of 60, 75, and 90 ppm, respectively. In addition, quenching experiments revealed that the photocatalytic process *via* GZnTi catalyst was controlled by highly reactive hydroxyl radicals (·OH). In the end, real petrochemical effluent was effectively treated under visible light irradiation and optimal operational conditions. The TOC_t_/TOC_0_ and COD_t_/COD_0_ removal ratio was reduced to 14% and 8% after 440 min. Therefore, the obtained results suggest that GZnTi has excellent photocatalytic abilities under visible light for decontamination of non-biodegradable compounds and real effluents.

## Conflicts of interest

There are no conflicts to declare.

## Supplementary Material

RA-008-C8RA07936F-s001
