# Supplementary material for: Photocatalytic decontamination of phenol and petrochemical wastewater through ZnO/TiO2 decorated on reduced graphene oxide nanocomposite: influential operating factors, mechanism, and electrical energy consumption
Source: RSC Adv. 2018 Nov 30;8(70):40035–53. doi: 10.1039/c8ra07936f (PMC9091293; doi:10.1039/c8ra07936f)

Electronic Supplementary Material (ESI) for RSC Advances.
This journal is © The Royal Society of Chemistry 2018

Figure S1. Adsorption of phenol on GZnTi within 100 min at temperature of 25.6 ℃, pH of 4, catalyst loading of 0.6 g/L and phenol concentration of 30 ppm.

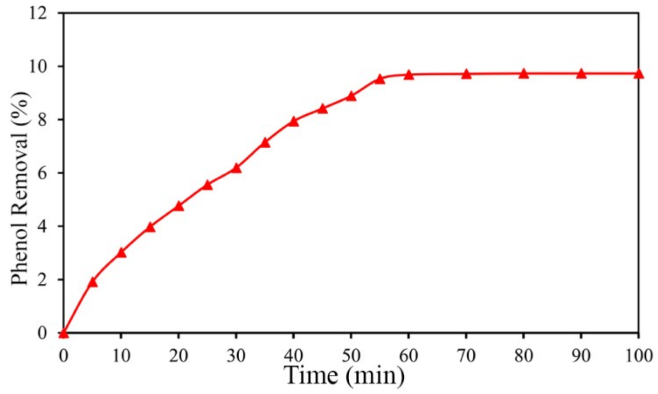

Supplement: RA-008-C8RA07936F-s001 [file RA-008-C8RA07936F-s001.pdf]
